# Supplementary material for: Quantification of different iron forms in the aceruloplasminemia brain to explore iron-related neurodegeneration
Source: Neuroimage Clin. 2021 Apr 3;30:102657. doi: 10.1016/j.nicl.2021.102657 (PMC8055714; doi:10.1016/j.nicl.2021.102657)
Supplement: Supplementary data 1 [file mmc1.docx]

**Supplementary materials**

**Bi-exponential echo decay in the basal ganglia**

A hint of deviation from a mono-exponential echo-decay was observed in the tissue block containing the basal ganglia. We note that in order to fit the eight-echo-decay with a model featuring four parameters, we performed a fit on at least six echoes above the noise level. A component was considered ‘true’ if its percentual fraction was at least 10% of the total amplitude decay and was discarded if the R_2_* ~ 0. The fast component was retained if R_2_*_fast_>3R_2_*_slow_ and if the coefficient of determination of the bi-exponential fit was larger than the one of the mono-exponential fit by at least the 0.1%.


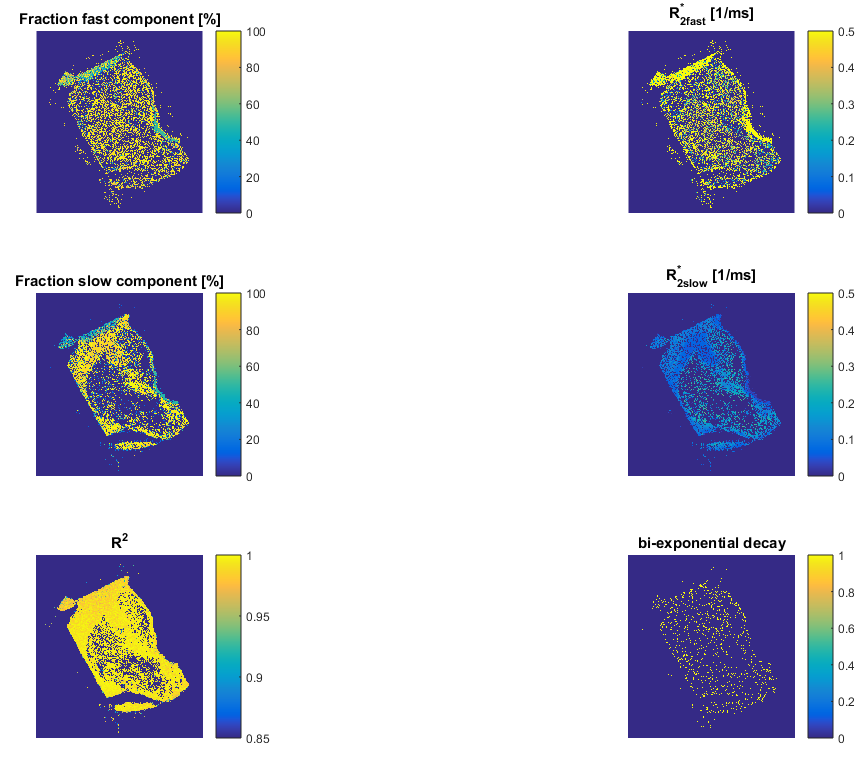


**Figure S1. Results of the bi-exponential fit on the tissue block containg the basal ganglia.** The plot titles refer to the fitting parameters of the model: y=A_fast_∙exp(-TE∙R_2_*_fast_)+ A_slow_∙exp(-TE∙R_2_*_slow_). The fractions were calculated in the following manner: Fraction_i_=[A_i_/( A_slow+_ A_fast_)] ∙100, where i=fast/slow.

Figure S1 shows that the fast decay is primarily localized in the gray matter structures, while the slow decay is confined in the white matter. The panel termed ‘bi-exponential decay’ shows the pixels where the exponential decay deviates from pure mono-exponential, given the assumptions above.

***Figure S2. Power attenuation experiment on the g’=4.3 band in one of the samples.*** *The amplitude peak-to-peak of the g’=4.3 band is shown as a function of the microwave power. The solid arrow shows the working point of the experiments presented in this work. Spectra were acquired at 6 K.*


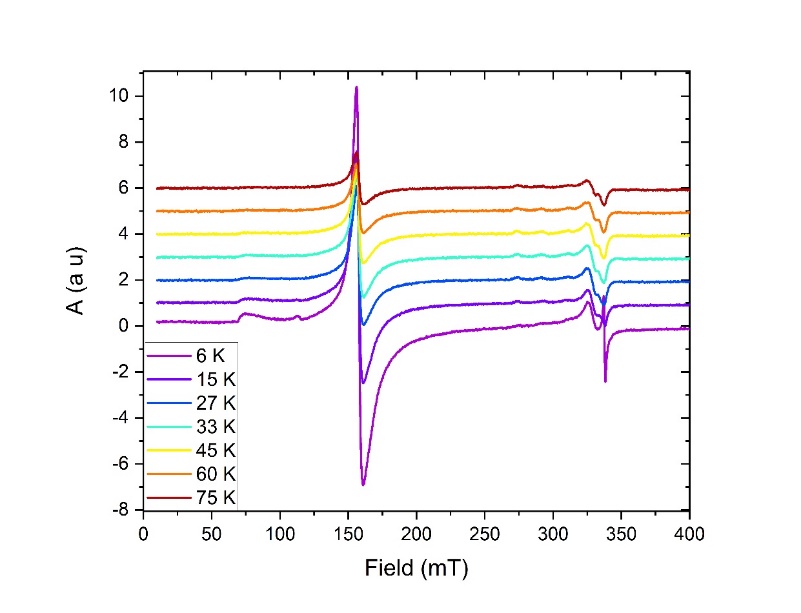


**Figure S3. Results of the temperature-dependent study of the putamen tissue block.** The left panel shows the spectra acquired at different temperatures in identical experimental conditions. The right panel shows the amplitude peak-to-peak of the g’=4.3 band (black circles) and the fit of the data to the Curie law (blue line) and the Curie-Weiss law (red line). The Curie-Weiss temperature extracted from the fit was -5.7 ±0.4 K.

| Fit of the EPR spectra  Baseline-corrected EPR spectra were fit in EasySpin to the Hamiltonian below:  $\hat{H}=g{\cdot\mu}_{B}\left( B\cdot S \right)+D\left( S_{z}^{2}-S\left( S+1 \right)/3 \right)+E\left( S_{x}^{2}-S_{y}^{2} \right)$  where g is the Landé factor, μ_B_ the Bohr magneton, B is the applied field and S the spin operator. The two final terms represent the zero-field splitting, where D is the axial splitting, and E the rhombic splitting.  An example of data fitting and best fitting parameters is reported below. The second integral of the fitted spectrum was used to estimate iron concentrations in this work.  *Table S1. Best fitting parameters of the high-spin Fe^3+^* *Hamiltonian* $\hat{\boldsymbol{H}}$*, for the medial division of the thalamus.* | | | |  |
| --- | --- | --- | --- | --- |
| g_x_, g_y_, g_z_ | D (MHz) | E/D | g_x_-strain, g_y_-strain, g_z_-strain | |
| 1.8046, 1.9816, 2.0102 | 20960 | 0.3324 | 1.1475, 0.0962, 0.0135 | |
| *The g-strain is used here phenomenologically to take into account the broadening of the line. Its high value shows that the actual line-broadening mechanism is probably a different factor, such as a distribution of D-values for example. Broad lines are expected, because the Fe^3+^* *complexes giving rise to this signal can be expected to have a distribution of ligand environments that give rise to different EPR parameters.* | | | | |

**Figure S4. EPR spectrum acquired on the medial division of the thalamus at 6 K (blue line).** Fit to the high-spin Fe^3+^ in red. The best fitting parameters are reported in Table S1.
